# Anti-inflammatory activity of d-pinitol possibly through inhibiting COX-2 enzyme: *in vivo* and *in silico* studies

**DOI:** 10.3389/fchem.2024.1366844

**Published:** 2024-04-16

**Authors:** Mst. Farjanamul Haque, Heba A. S. El-Nashar, Md. Showkoth Akbor, Mohammed Alfaifi, Mehedi Hasan Bappi, Afsana Kabir Chowdhury, Muhammad Kamal Hossain, Mohamed El-Shazly, Tala Albayouk, Na’il Saleh, Muhammad Torequl Islam

**Affiliations:** ^1^ Department of Pharmacy, Bangabandhu Sheikh Mujibur Rahman Science and Technology University, Gopalganj, Bangladesh; ^2^ Department of Pharmacognosy, Faculty of Pharmacy, Ain Shams University, Cairo, Egypt; ^3^ Department of Clinical Laboratory Sciences, College of Applied Medical Sciences, King Khalid University, Abha, Saudi Arabia; ^4^ Department of Pharmacy, East West University, Dhaka, Bangladesh; ^5^ School of Pharmacy, Jeonbuk National University, Jeonju, Republic of Korea; ^6^ Department of Pharmacy, University of Science and Technology Chittagiong, Chittagong, Bangladesh; ^7^ Department of Chemistry, College of Science, United Arab Emirates University, Al Ain, United Arab Emirates; ^8^ Pharmacy Discipline, Khulna University, Khulna, Bangladesh; ^9^ BioLuster Research Center, Dhaka, Bangladesh

**Keywords:** d-pinitol, paw edema, anti-inflammatory effect, cyclooxygenase-2, molecular docking study

## Abstract

**Introduction:** D-pinitol, a naturally occurring inositol, has diverse biological activities like antioxidant, antimicrobial and anticancer activities. This study aimed to evaluate anti-inflammatory effect of d-pinitol in a chick model. Additionally, *in silico* studies were performed to evaluate the molecular interactions with cyclooxygenase-2 (COX-2).

**Methods:** The tested groups received d-pinitol (12.5, 25, and 50 mg/kg) and the standard drugs celecoxib and ketoprofen (42 mg/kg) via oral gavage prior to formalin injection. Then, the number of licks was counted for the first 10 min, and the paw edema diameter was measured at 60, 90, and 120 min.

**Results and Discussion:** The d-pinitol groups significantly (*p* < 0.05) reduced the number of paw licks and paw edema diameters, compared to negative control. When d-pinitol was combined with celecoxib, it reduced inflammatory parameters more effectively than the individual groups. The *in silico* study showed a promising binding capacity of d-pinitol with COX-2. Taken together, d-pinitol exerted anti-inflammatory effects in a dose-dependent manner, possibly through COX-2 interaction pathway.

## 1 Introduction

Inflammation is a biological response of the immune system to harmful stimuli, such as pathogens, damaged cells, or irritants (Aghasafari et al., 2019). It is a complex process involving various cells, chemicals, and signaling pathways that work together to remove the offending agent and repair any tissue damage (Cruvinel Wde et al., 2010). During inflammation, the affected area may become red, swollen, painful, and warm as a result of increased blood flow and the accumulation of immune cells and fluids (Turner et al., 2014). In some cases, inflammation can be beneficial and necessary for healing and protection, such as in response to a cut or infection (Liu et al., 2021). However, if inflammation persists or becomes chronic, it can lead to tissue damage, impaired function, and various diseases, such as arthritis, diabetes, heart disease, or cancer (Furman et al., 2019). In some cases, inflammation is intolerable and affects a person’s health. Therefore, controlling and balancing inflammation is important for maintaining health and preventing chronic conditions. Inflammation can be caused by a variety of factors, including infections, injuries, environmental factors, autoimmune disorders, chronic stress, and an unhealthy lifestyle (Liu et al., 2021).

Inflammation involves the activation of the cyclooxygenase (COX) enzyme, which plays a key role in the synthesis of prostaglandins (Ricciotti and FitzGerald, 2011). Prostaglandins are hormone-like substances that are involved in a wide range of physiological processes, including inflammation, pain, and fever (Coulthard et al., 2012). There are two types of COX enzymes: COX-1 and COX-2. COX-1 is constitutively expressed in many tissues and is involved in maintaining normal physiological functions, such as regulating the production of mucus in the stomach lining, which helps to protect it from damage caused by stomach acid, regulating blood flow to the kidneys, and promoting platelet aggregation for blood clotting (Patrignani and Patrono, 2015; Faki and Er, 2021). COX-2, on the other hand, is induced during inflammation and is responsible for the synthesis of prostaglandins that promote inflammation, pain, and fever (Grösch et al., 2017). The COX enzymes catalyze the conversion of arachidonic acid, a type of fatty acid, into prostaglandins. Prostaglandins can then act on nearby cells to induce inflammation and increase blood flow to the affected area. They also sensitize nerve endings to pain, resulting in the characteristic pain associated with inflammation (Salvemini et al., 2013).

There are several drugs commonly used to treat inflammation, including nonsteroidal anti-inflammatory drugs (NSAIDs), glucocorticoids, and disease-modifying antirheumatic drugs (DMARDs) (Bermas, 2014). Non-selective NSAIDs, such as aspirin, ibuprofen, and naproxen, work by inhibiting both COX-1 and COX-2 enzymes, which reduces the production of prostaglandins and reduces inflammation, pain, and fever (Jahnavi et al., 2019). However, non-selective NSAIDs irritate the stomach lining and cause inflammation, which can lead to ulcers, bleeding, and perforation of the stomach or intestines (Bjarnason et al., 2018). Long-term use of these drugs can cause side effects, including kidney damage and an increased risk of heart attack and stroke (Day and Graham, 2013). Selective NSAIDs, such as celecoxib, inhibit cox-2 enzymes, which relieve pain and inflammation in conditions such as osteoarthritis, rheumatoid arthritis, and acute pain (Gong et al., 2012). It can cause side effects such as stomach upset, diarrhea, headache, dizziness, and an increased risk of cardiovascular events (Auriel et al., 2014). A Cox-2-selective anti-inflammatory like Rofecoxib (Vioxx)^®^ was voluntarily withdrawn from the market in 2004 due to an increased risk of cardiovascular events, including heart attack and stroke (Stiller and Hjemdahl, 2022). Valdecoxib (Bextra)^®^ was also withdrawn from the market in 2005 due to an increased risk of serious skin reactions and cardiovascular events (Atukorala and Hunter, 2013). Long-term use of glucocorticoids can cause side effects including weight gain, high blood pressure, diabetes, weakened bones, and an increased risk of infection (Oray et al., 2016). DMARDs can have side effects, including liver and kidney damage, infections, and an increased risk of certain cancers (Solomon et al., 2020; Akbor et al., 2023b).

Natural products contain a distinctive chemical variety that leads to variability in their biological properties and drug-like qualities (Yuliana et al., 2011; Abd El-Ghffar et al., 2017; El-Nashar et al., 2021). This chemical diversity has evolved over millions of years via natural selection (Abdelghffar et al., 2022; El-Nashar et al., 2022a; Askar et al., 2022). These goods have emerged as one of the most crucial sources for creating fresh lead compounds and scaffolds (El-Shawi et al., 2023; Younis et al., 2023). The ongoing use of natural goods will help address the pressing requirement for developing efficient medications (El-Nashar et al., 2022b; Bhuia et al., 2023). Many natural compounds have been found to have pharmacological properties and are used as the basis for many drugs used to treat various diseases due to their low side effects, easy availability, and cost effectiveness (Yuan et al., 2016; Rabie et al., 2023). Natural agents such as curcumin, ginger, omega-3 fatty acids, resveratrol, and boswellia have been found to have anti-inflammatory properties (Maroon et al., 2010).

D-pinitol (DPL) is a naturally occurring, pharmacologically active molecule that is often a member of the significant inositol family. Pinitol is a 3-*O*-methyl-D-chiroinositol (Dube et al., 2020). It was first identified in Sugar pine, however it may be found and isolated from a variety of plants (Srivastava et al., 2020). It is a low-molecular-weight sugar-like molecule that is distinct from glucose, fructose, sucrose, or myo-inositol and exhibits lipophilic properties, was extracted from the ethanol extract of *Acacia nilotica* and the leaves of *Colutea cilicica* (Chaubal et al., 2005; Eser et al., 2017). However, members of the Leguminosae family are the major natural source of this compound and the pinitol was found from many other families Asteraceae, Pinaceae, Zygophyllaceous, Cupressaceae, Caryophyllaceae, Sapindaceae and Aristolochiaceae (Srivastava et al., 2020). DPL possesses many therapeutic properties, such as a diuretic effect, anxiolytic-like effects, anticonvulsant activity, antidepressant-like effects, sedative and locomotor effects, anti-diabetic effect/anti-hyperglycemic activity, memory enhancer, wound healing activity, antidiarrheal activity, anti-inflammatory activity, anti-nociceptive activity, antimicrobial activity, and anticancer activity (Srivastava et al., 2020). Previous study demonstrated that d-pinitol express anti-inflammatory activity in *in-vitro* study through inhibiting protein expression of Cox-2 in K562 cell (Eser et al., 2017).

The aim of this study is to evaluate the anti-inflammatory activity of DPL through *in-vivo* testing. Further *in silico* studies were carried out to find out whether DPL exhibits its anti-inflammatory activity through inhibiting the COX enzyme.

## 2 Materials and methods

### 2.1 Chemicals and reagents

**FIGURE 1:**
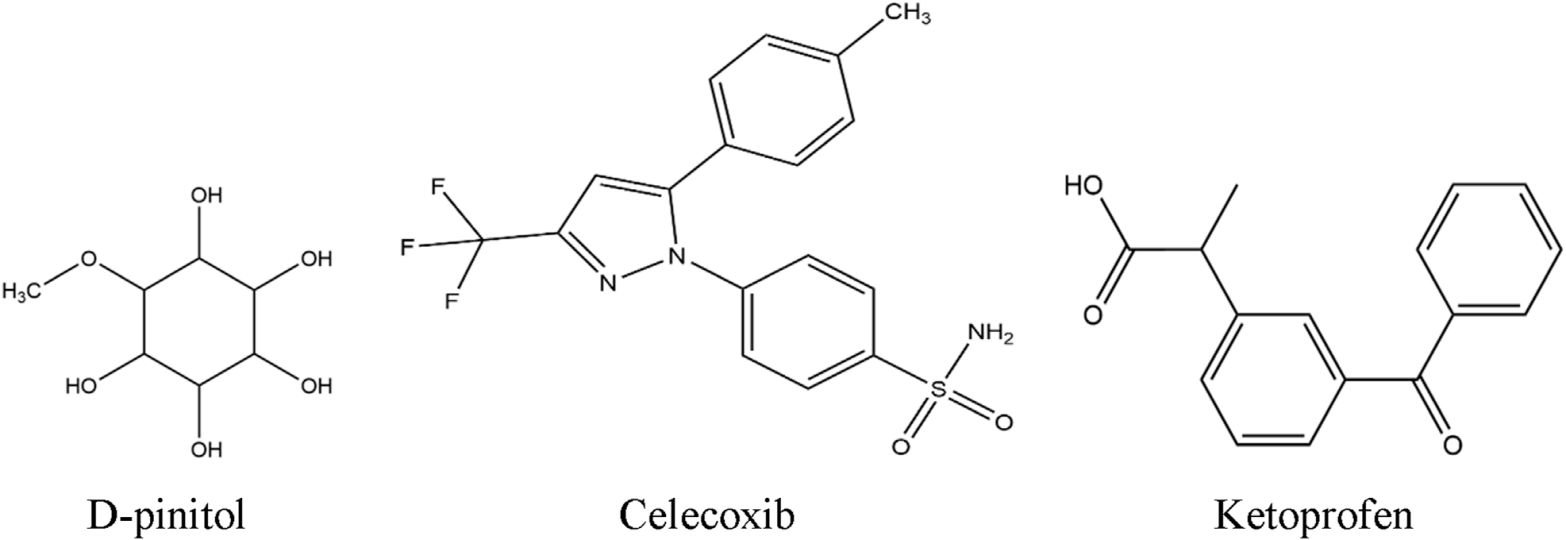
Chemical structure of reference drugs (d-pinitol, celecoxib and ketoprofen).

DPL was purchased from Sigma-Aldrich (Germany), while tween-80 and formaldehyde were bought from Merck India. Celecoxib and ketoprofen were purchased from the East-west Pharma (India) Pvt. Ltd., and Albion Laboratories Ltd. (Bangladesh), respectively. The chemical structures of these chemicals are illustrated in Figure 1.

### 2.2 Experimental animals

Chicks (40–42 g) of 2-days-old purchased from Nourish Grand Parent Ltd., Rangpur, Bangladesh and were maintained at Pharmacology Lab of Bangabandhu Sheikh Mujibur Rahman Science and Technology University (BSMRSTU), Gopalganj for the present study. The animals were allowed free access to standard food and water *ad libitum*. They were kept under controlled lighting (12 h dark/light cycle) at 27 ± 1°C until the test commenced. The present experiment was conducted from 08:00 a.m. to 3:00 p.m.; and the animals were monitored for 17 h to check their possible mortality after the study. This project was funded and approved by the Research Center of BSMRSTU (#2023-33) and approval.

### 2.3 Study design (formalin-induced paw edema in chicks)

For this study, we used 2-h fasted animals after 2-day acclimation in the laboratory environment. Briefly, a total of forty chicks (48–55 g, b.w.) of either sex were randomly distributed into eight different groups each containing five animals (Table 1). All the treatments were given via oral gavage 30 minutes prior to injection of formalin (0.5% formaldehyde) solution in the sub-plantar area of the right hind paw of the animals. 20 μL of formalin solution prepared in normal saline was injected into each animal (Woode et al., 2009; Fadel and Mustafa, 2023). Then the licking behavior within was counted for first 10 min and the paw edema diameter was measured by using a slide calipers at 60, 90 and 120 min after formalin injection. Paw edema was measured in comparison to the baseline (normal) paw diameter of each animal. Then the following parameters were determined.

**TABLE 1:** Name of treatment groups, composition, their dose, and biological targets.

Treatment group	Composition	Dose	Target receptor
Gr-I	Vehicle (0.5% Tween 80 dissolved in normal saline)	10 mL/kg	-
Gr-II	D-pinitol (DPL)	12.5 mg/kg	Under investigation
Gr-III		25 mg/kg	
Gr-IV		50 mg/kg	
Gr-V	Celecoxib (CXB)	42 mg/kg	COX2
Gr-VI	Ketoprofen (KFN)	42 mg/kg	COX1
Gr-VII	DPL-25 + CXB-42	25 mg/kg + 42 mg/kg	Under investigation
Gr-VIII	DPL-25 + KPN-42	25 mg/kg + 42 mg/kg	Under investigation

All treatments are given at 10 mL/kg via oral gavage (p.o.)

Diameter of paw edema (mm) = Paw diameter in observed time–Baseline paw diameter

$$\%\text{reduction of paw edema}=\left[\left({\text{PDOT}}_{\text{Vehicle}}\;–\;{\text{PDOT}}_{\text{Test}}\right)÷{\text{PDOT}}_{\text{Vehicle}}\right]\times 100$$

Where, PDOT means paw diameter in observed time.

### 2.4 *In silico* study

#### 2.4.1 ADME analysis

Pharmacokinetic properties of DPL determine how drugs are absorbed, distributed, metabolized, and eliminated in the body (Honório et al., 2013). Canonicals SMILES ID: COC1C(C(C(C(C1O)O)O)O)O were collected from PubChem (PubChem ID: 164619) and ADME properties were analyzed by using SwissADME online tool (http://www.swissadme.ch/) (Akbor et al., 2023a).

#### 2.4.2 Preparation of ligand structure

The preparation of ligand structures for docking studies involved selecting three ligands, namely, DPL, CXB, and KFN. The ligand structures were retrieved from the PubChem database and converted to 3D format using Chem3D software. The energy minimization of the ligand structures was performed using the MM2 force field in Chem3D software (version Pro21.0 PerkinElmer Informatics, Inc., Boston, MA, United States) (Akbor et al., 2023a). Finally, DPL structure was validated using Lipinski’s rule of five and ADMET analysis in the SwissADME online tool (Bappi et al., 2023b). This ensured that the ligands had favorable pharmacological properties and were suitable for molecular docking studies with the prepared protein structures.

#### 2.4.3 Preparation of protein structure

Protein structures for docking studies involved COX-1 and Cox-2. The protein sequences were retrieved from the UniProt database. The crystal structures of COX-1 and COX-2 were retrieved from the Protein Data Bank (PDB) (Zong et al., 2018; Fan et al., 2024). The prepared protein structures were then processed using BIOVIA Discovery Studio (version 21.1.0, Dassault, Systemes, France) to remove water molecules, ligands, and other heteroatoms and add hydrogen atoms. Energy minimization was performed using Swiss-PDB Viewer (version 4.1.0, Swiss Institute of Bioinformatics (SIB), Basel, Switzerland) software, and the structures were validated using PROCHECK in the SAVES (version 6.0) online tool. The final prepared protein structures were used for molecular docking studies with the ligand structures.

#### 2.4.4 Docking protocols

The molecular docking of ligands with target proteins was carried out using PyRx software (version 0.8.0.0, The Scripps Research Institute, La Jolla, CA, United States) (Bappi et al., 2024). Input files for docking were prepared by setting appropriate docking algorithms and parameters. Ligands were docked with prepared protein structures, and the docking results were analyzed using PyMOL (version 2.5.5, Schrodinger Inc., New York, NY, United States) and BIOVIA Discovery Studio (Mia et al., 2023; Prottay et al., 2024). The docking analysis involved the visualization of ligand-protein interactions, the calculation of binding energy and other parameters, and the identification of potential binding sites and key amino acid residues. The docking results provided insights into the binding modes and affinity of the ligands towards the target proteins and were used to guide further *in vivo* studies.

### 2.5 Statistical analysis

The results are presented as Mean ± S.E.M. (standard error of mean) or percentage values. The data are analyzed by means of analysis of variance (ANOVA) followed by *t*-Student–Newman–Keuls’s as *post hoc* test using the statistical software GraphPad Prism (version 6.5) and experimental groups are compared with the vehicle (control) group. The levels statistical significance ranged with *p* < 0.05 at 95% confidence intervals (Bappi et al., 2023a).

## 3 Results and discussion

### 3.1 *In vivo* study

Several inflammatory mediators, including pro- and anti-inflammatory mediators, are synthesized and released during inflammatory reactions of various sorts (Nkadimeng et al., 2021). Among the inflammatory mediators and cellular pathways that have been thoroughly investigated in connection with human pathological conditions are cytokines (such as interferons, interleukins, and tumor necrosis factor (TNF)), chemokines (such as monocyte chemoattractant protein 1), eicosanoids (such as prostaglandins and leukotrienes), and the potent transcription factor nuclear factor B that modulates inflammation. A few mediators, including interleukin (IL)-12, have both pro- and anti-inflammatory effects (Harizi and Gualde, 2006). In our *in-vivo* studies, the lowest number of licks (9.33 ± 1.08) were observed when DPL was administered at 50 mg/kg compared to NC and the standard drugs CXB and KPN. Gradually reducing the dose of DPL, we observed that the number of licks was increasing. On the other hand, combined administration of DPL 25 mg/kg and celecoxib 42 mg/kg showed the lowest number of licks (6.67 ± 1.47) than all other groups, while the DPL-25 +KPN-42 combine groups showed a number of licks (9.67 ± 2.94) (Table 2). There were significant changes in edema in different groups, and the changes in edema varied with time. The negative control group showed the maximum edema of all other groups. DPL at 50 mg/kg showed lower edema than DPL-25, NC, CXB, and KPN. When DPL was administered, there was a gradual decrease in edema at 60, 90, and 120 min. We also observed significant lowest edema and decreasing with time in the combined groups of DPL-25+CXB-42 and DPL-25+KPN-42 (Table 2). The DPL 50 mg/kg group showed the maximum percentage of edema reduction with time compared to negative control. The percentage of reduction edema gradually increases with time. The combined group also showed a significantly higher percentage of edema reduction than all other groups. Combine group DPL and celecoxib exhibit maximum percentage of reduction edema (Table 3). K562 cell lines were treated with DPL, which was isolated from *C. cilicica* leaves, to reduce inflammation (Eser et al., 2017). It was shown that DPL at doses (10, 20, 40 mg/kg, p. o.) substantially decreased pro-inflammatory cytokines TNF, IL-6, and IL-1 levels in mice in research on how DPL attenuates cisplatin-induced nephrotoxicity in rats (Vasaikar et al., 2018). Previous studies revealed that DPL exhibits anti-inflammatory activity via suppression of the NF-κB pathway (Kumar et al., 2004). In a different study, the amount of granuloma formation caused by the implantation of cotton pellets was marginally reduced. Synergistic anti-inflammatory effects of pinitol and glucosamine in rats revealed that 20 mg/kg of pinitol considerably inhibited paw edema caused by the injection of carrageenan (Kim et al., 2005). (+)-pinitol, derived from *Abies pindrow* leaves, has shown strong anti-inflammatory activity in carrageenin-induced paw edema in rats (Singh et al., 2001). In our *in-vivo* study, we observed that DPL showed anti-inflammatory activity in a dose-dependent manner. The lowest numbers of licking and maximum reduction of edema were observed when DPL was administered at a 50 mg/kg dose, compared to the negative control, CXB, and KFN. We also observed that edema was significantly reduced with time (60 min, 90 min, and 120 min) when chicks were administered DPL at 25 or 50 mg/kg administered by DPL at 25 or 50 mg/kg.

**TABLE 2:** Paw licking and paw edema diameter profiles observed in test and/or control groups.

Treatment groups	Licking	Edema diameter (mm)		
		60 min	90 min	120 min
Gr-I	48.93 ± 3.58	2.85 ± 0.25	2.75 ± 0.15	2.60 ± 0.20
Gr-II	17.33 ± 4.81^*^	2.80 ± 0.00	2.00 ± 0.40^*^	1.33 ± 0.41^*^
Gr-III	14.67 ± 1.63^*^	2.48 ± 0.30^*^	1.75 ± 0.31^*^	0.92 ± 0.01^*b^
Gr-IV	9.33 ± 1.08^*abc^	2.00 ± 0.41^*abc^	1.56 ± 0.30^*a^	0.67 ± 0.41^*abc^
Gr-V	12.78 ± 3.38^*a^	2.40 ± 0.30^*^	1.60 ± 0.20^*a^	1.00 ± 0.40^*^
Gr-VI	10.67 ± 2.72^*ab^	2.20 ± 0.30^*ab^	1.50 ± 0.30^*ab^	0.80 ± 0.20^*ab^
Gr-VII	6.67 ± 1.47^*abc^	2.00 ± 0.00^*abc^	1.50 ± 0.20^*ab^	0.90 ± 0.00^*b^
Gr-VIII	9.67 ± 2.94^*ab^	2.17 ± 0.20^*ab^	1.50 ± 0.30^*ab^	0.67 ± 0.20^*abc^

Values are Mean ± SEM (standard error of mean) (n = 5); One-way ANOVA, followed by t-Student–Newman–Keuls’s as post hoc test; *p < 0.05 compared to the Vehicle group; ^a^ *p* < 0.05 compared to the Gr-III; ^b^ *p* < 0.05 compared to the Gr-V; ^c^ *p* < 0.05 compared to the Gr-VI; Gr-I: vehicle; Gr-II: D-pinitol (DPL) 12.5 mg/kg; Gr-III: DPL, 25 mg/kg; Gr-IV: DPL, 50 mg/kg; Gr-V: Celecoxib (CXB) 42 mg/kg; Gr-VI: Ketoprofen (KPN) 42 mg/kg; Gr-VII: DPL-25 + CXB-42; Gr-VIII: DPL-25 + KPN-42.

**TABLE 3:** Percentage reduction of paw edema in test and/or standard groups when compared to the control group.

Treatment groups	Reduction edema (%)		
	60 min	90 min	120 min
Gr-II	0.00	27.27	48.85
Gr-III	12.98	36.36	64.62
Gr-IV	29.82	43.27	74.23
Gr-V	15.79	65.71	61.54
Gr-VI	22.81	45.45	69.23
Gr-VII	29.82	45.45	65.38
Gr-VIII	23.86	45.45	74.23

Values are expressed as percentage in comparison to the vehicle group; Gr-II: D-pinitol (DPL) 12.5 mg/kg; Gr-III: DPL, 25 mg/kg; Gr-IV: DPL, 50 mg/kg; Gr-V: Celecoxib (CXB) 42 mg/kg; Gr-VI: Ketoprofen (KPN) 42 mg/kg; Gr-VII: DPL-25 + CXB-42; Gr-VIII: DPL-25 + KPN-42.

### 3.2 *In silico* study

#### 3.2.1 ADME properties of d-pinitol

**FIGURE 2:**
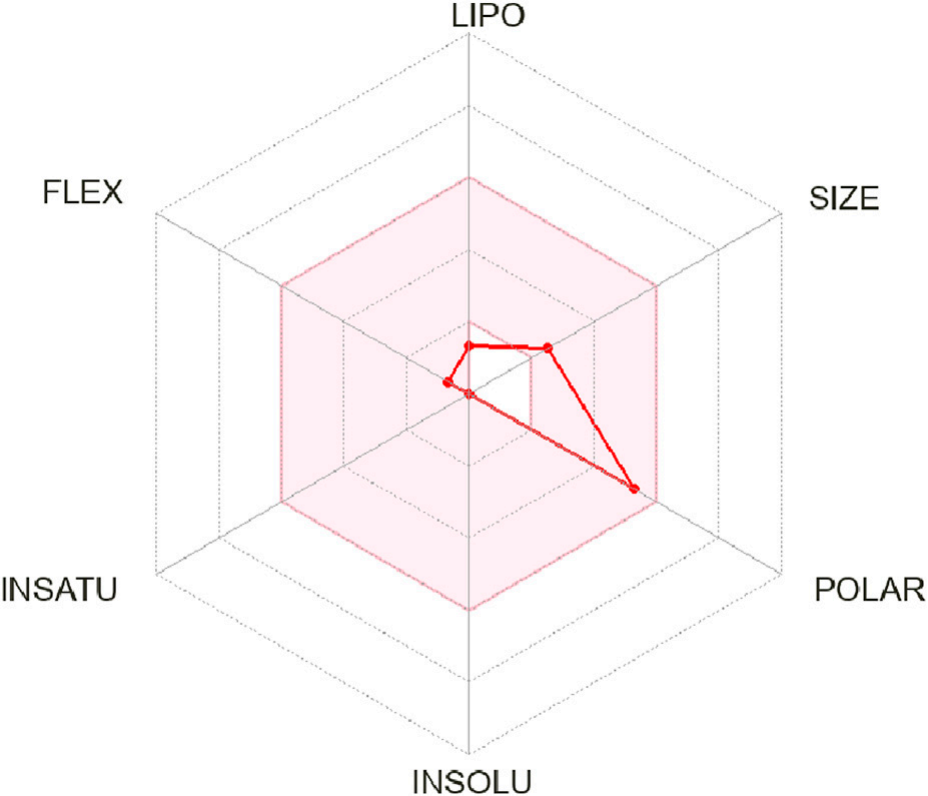
Bioavailability reader of d-pinitol.

In ADME analysis of DPL, we observed that DPL had five H-bond donors and six H-bond acceptors, with a molecular weight of 194.18 g/mol. The consensus log P_o/w_ value for DPL was −2.26, indicating low lipophilicity, all of which are within the limits set by Lipinski’s Rule of Five. The water solubility of DPL is high, with a Log S (ESOL) value of 1.02 and a solubility of 2.03 ± 03 mg/mL (1.05 ± 01 mol/L), indicating high water solubility. DPL had a high molar refractivity value of 40.45, indicating its potential to interact with polar molecules. The compound is slowly absorbed by the gastrointestinal tract and is not blood-brain barrier (BBB)-permeable. The bioavailability score for DPL was 0.55, indicating moderate bioavailability as shown in Figure 2. DPL exhibited poor skin permeation, with a log Kp value of −9.74 cm/s (Table 4).

**TABLE 4:** Physiochemical, pharmacokinetics and drug likeness properties of d-pinitol.

Parameters	Value
H-bond donors	5
H-bond acceptors	6
Molecular weight	194.18 g/mol
Lipophilicity (Consensus log P_o/w_)	−2.26
Water solubility	Log *S* (ESOL): 1.02
	Solubility: 2.03e+03 mg/mL; 1.05e+01 mol/L
	Highly Soluble
Molar Refractivity	40.54
GI absorption	Low
BBB permeant	No
Bioavailability Score	0.55
Log *K*_p_ (skin permeation)	−9.74 cm/s

#### 3.2.2 Interaction of d-pinitol and ketoprofen with COX-1

**FIGURE 3:**
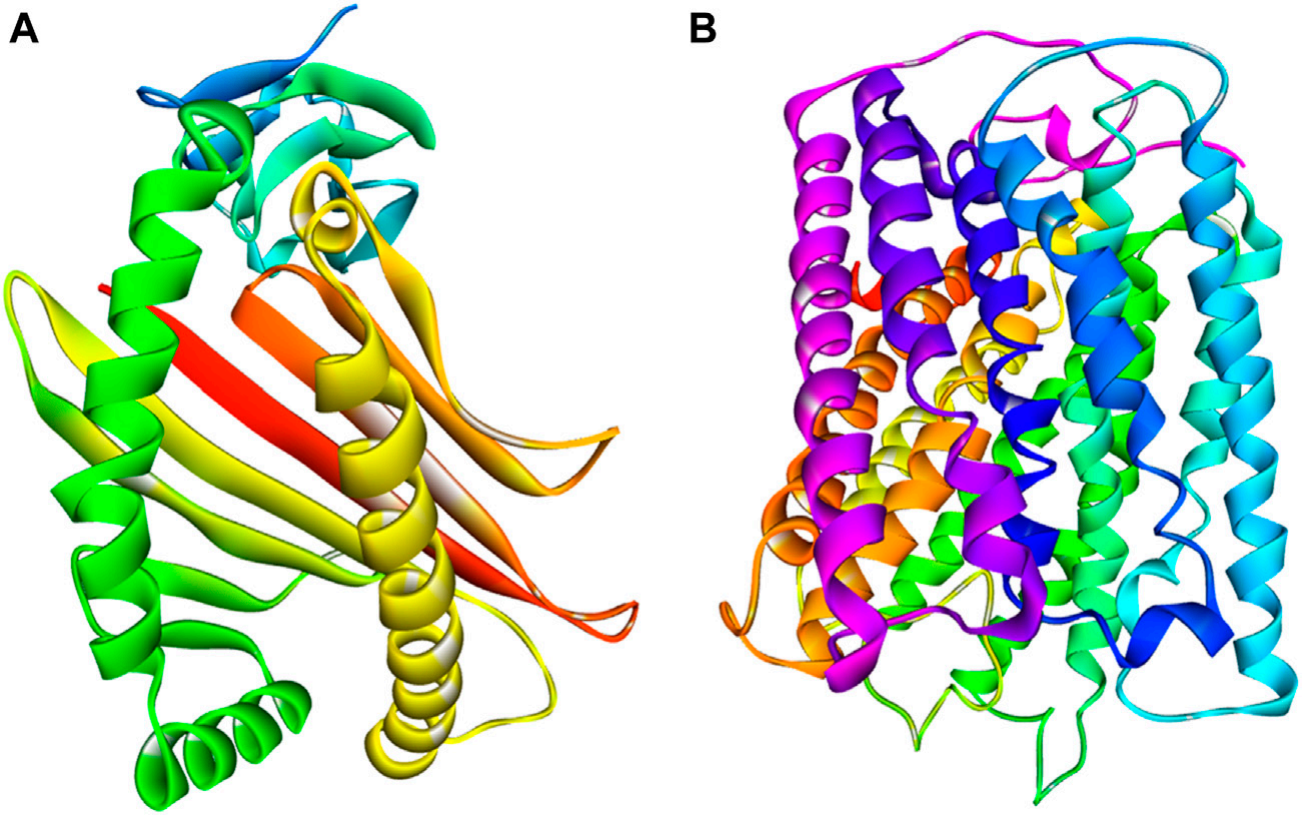
3D crystal structure of proteins **(A)** COX-1 **(B)** COX-2.

It seems implausible that COX-2 is exclusively responsible for escalating the inflammatory response by producing pro-inflammatory prostaglandins (Li et al., 2022; Islam et al., 2023). In the inflammatory process, COX-2 is a crucial enzyme that catalyzes the rate-limiting stages in the conversion of arachidonic acid to prostaglandins (Cao and Prescott, 2002). Selective COX-2 inhibitor therapy has previously been recognized as an effective strategy for the management of inflammation with minimal side effects (Stichtenoth and Frölich, 2003). The most often prescribed drugs now for the treatment of inflammatory illnesses by targeting COX-2 are both traditional and modern NSAIDs (Everts et al., 2000). Finding appropriate substitutes for these routinely given medications, however, is a hot topic in medicinal chemistry research because of the NSAIDs’ negative cardiovascular side effects (Desai et al., 2018). Here, macromolecules or Proteins COX-1 and COX-2 of *Homo sapiens* (*UniProt* IDs: P00395 and P00403) were collected from the PDB database (PDB ID: 5z62). The 3D crystal structures of subdivisions COX-1 and COX-2 were retrieved from the same protein, human cytochrome C oxidase (Figure 3). The A and B chains of these proteins indicate COX-1 and COX-2, respectively. COX-1 contains four helices and 513 amino acid residues, while COX-2 contains 16 helices and 227 amino acid residues. Their 3D crystal structure validity was rechecked by procheack (Kamli et al., 2023).

**FIGURE 4:**
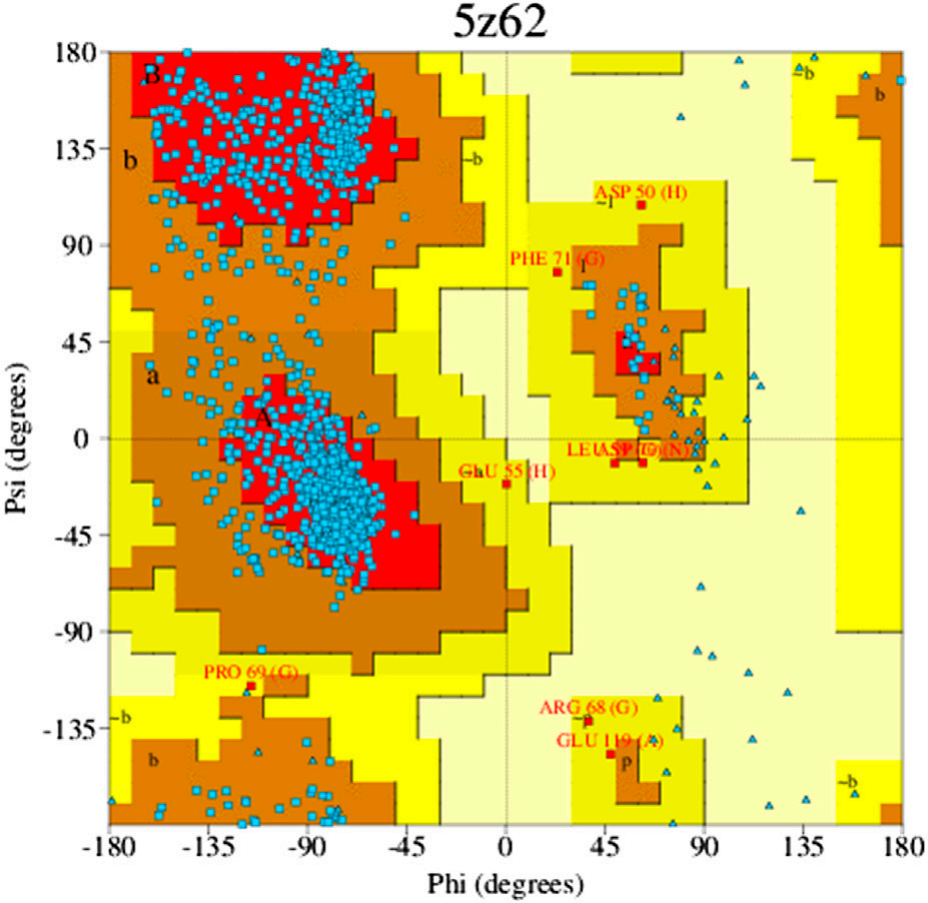
Favorable zone of 5z62 protein.

The Ramachandran plot is a way to evaluate the quality of the protein structure. It is a graphical representation of the torsion angles (phi and psi) of the amino acid residues in a protein, which shows the allowed and disallowed regions of the conformational space. The allowed regions correspond to energetically favorable conformations, while the disallowed regions correspond to energetically unfavorable conformations. The color and shade of the map indicate the various components described; those “core” regions, or dark regions (shown in red), correspond with the most favorable Φ-Ψ value combination. In a perfect world, these “core” sections would include more than 90% of the leftover. One of the most consistent indicators of stereo chemical integrity is the fraction of residues situated in “core” areas (Figure 4). The residues in the most favorable areas for 5z62 proteins range approximately 87.8% according to Ramachandran plot estimates.

#### 3.2.3 Interaction of d-pinitol and ketoprofen with COX-1

**FIGURE 5:**
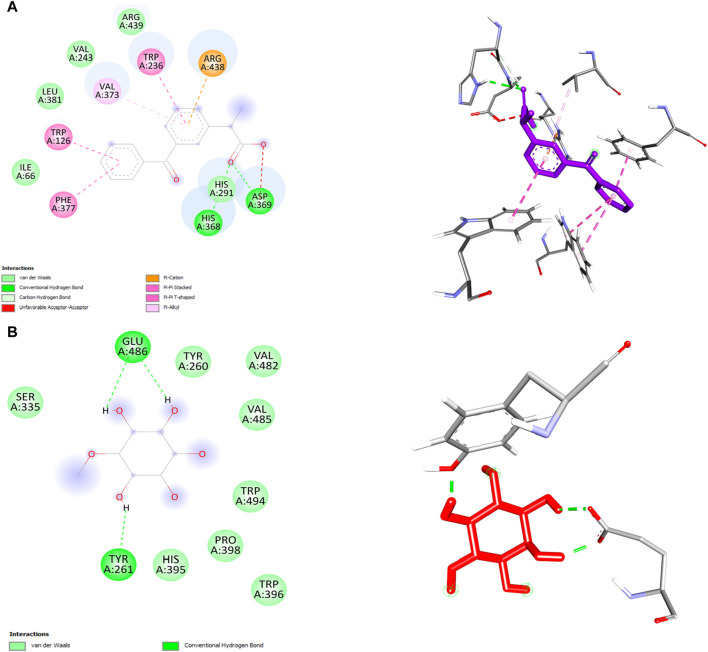
2D and 3D structure of ligand and COX-2 interaction. **(A)** ketoprofen **(B)** d-pinitol.

In this study, we performed molecular docking analysis to investigate the potential binding affinity of DPL and KFN with COX-1. The results of the molecular docking analysis showed that KFN had a strong binding affinity of −9.0 kcl/mol with COX-1, while DPL showed a binding affinity of −5.4 kcal/mol with COX-1. The molecular docking analysis also revealed that DPL formed three conventional hydrogen bonds. KFN interacts with specific residues in COX-1 through seven hydrogen bonds and five other types of bonds. KFN formed a hydrogen bond with HIS368, ASP369, and HIS368 residues of COX-1. KFN also formed one pi-cation bond, one pi-anion bond, one pi-pi stacked bond, and two pi-pi T-shaped bonds with residues of COX-1 (Table 5 and Figure 5).

**TABLE 5:** Ketoprofen and d-pinitol best binding affinity values and non-bond interactions with COX-1.

Ligand	Binding affinity (Kcal/mol)	No. of bond	H-bond			Other bond		
			Residues	Types	Bond length (Å)	Residue	Types	Bond length (Å)
Ketoprofen	−9.0	9	HIS368, ASP369, HIS368	Conventional, Conventional, Carbon	2.62945 2.13376 3.53604	ARG438, TRP126, TRP436, PHE377, TRP236, VAL373	Pi-Cation	3.56391 4.94106 4.80284 3.79799 5.1302 5.48081
							Pi-Pi Stacked	
							Pi-Pi Stacked	
							Pi-Pi Stacked	
							Pi-Pi T-shaped	
							Pi-Alkyl	
D-pinitol	−5.4	3	GLU486, TYR261, GLU486	Conventional, Conventional, Conventional	2.15671 1.73546 1.9094	-	-	-

#### 3.2.4 Interaction of d-pinitol, celecoxib, and ketoprofen with COX-2

**FIGURE 6:**
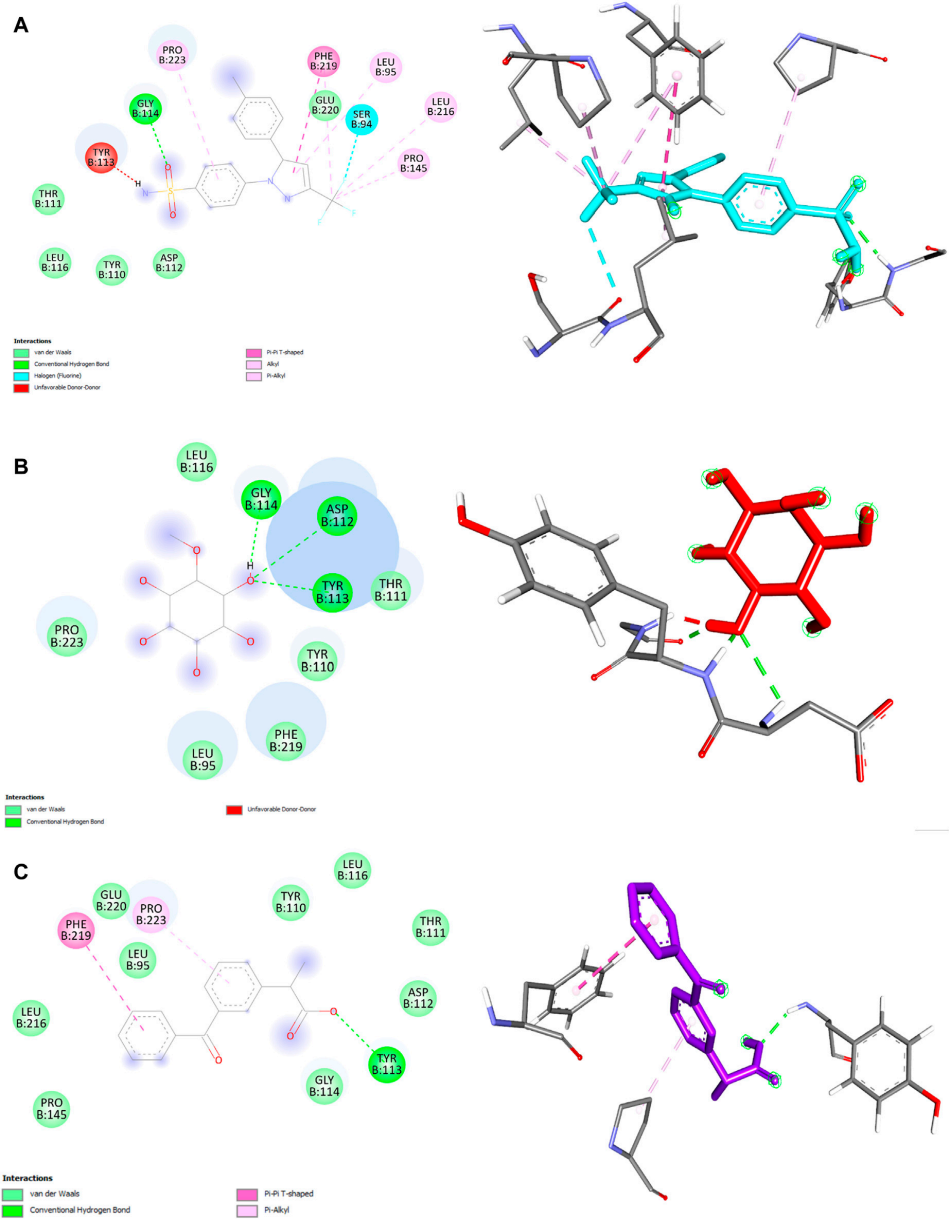
2D and 3D structure of ligand and COX-2 interaction. **(A)** Celecoxib **(B)** d-pinitol **(C)** ketoprofen.

We also performed molecular docking analysis to investigate the potential binding affinity of DPL, CXB, and KFN with COX-2. The analysis revealed that DPL, CXB, and KFN showed favorable binding affinity of −5.0, −7.7, and −7.5 kcl/mol, respectively, with COX-2. CXB interacts with COX-2 through eight bonds, including two conventional hydrogen bonds with ASN77, TRP147 residue, and ten other bonds. KFN interacts with COX-2 through one conventional H-bond, three pi-sigma bonds, one pi-pi-stacked bond, and one pi-pi-T-shaped bond. We also observed that DPL interacts with COX-2 through two hydrogen bonds with ASN77 and one p-sigma bond with TYR123 residues (Table 6). There were some similar residues among DPL and COX-2 interaction pocket residues (Figure 6). In DPL and COX-2 binding interactions, COX-2 pocket residues are GLY114, TYR113, ASP112, THR111, TYR110, PHE219, LEU95, PRO223, and LEU116. In celecoxib and COX-2 binding interactions, COX-2 pocket residues are GLY114, TYR113, ASP112, THR11, TYR110, PHE219, LEU95, PRO223, LEU 116, GLU220, SER94, PRO145, and LEU216 (Figure 6). According to previous reports, several naturally occurring substances have the ability to suppress COX-2, which has positive effects on inflammation and certain types of cell damage. Scientists observed DPL impact on COX-2 in colitis-affected animals. By employing PCR tests, the relative levels of COX-2 were found, the mRNA levels and protein expression of COX-2 were decreased following DPL therapy, as demonstrated by the Western blot analysis of COX-2 protein expressions (Lin et al., 2021). TNF, ILs, COX II, and vascular endothelial growth factor expression is downregulated by DPL, which is how it exerts its anti-arthritic properties (Yang et al., 2021) Researchers found that DPL suppressed the protein production of Cox-2 in K562 cells after stimulating the cells with it. Additionally, researchers saw a decrease in Cox-2 protein expression when non-cytotoxic DPL doses were used (Eser et al., 2017).

**TABLE 6:** Celecoxib, ketoprofen and d-pinitol best binding affinity values and non-bond interactions with COX-2.

Ligands	Binding affinity (Kcal/mol)	No. of bond	H-bond			Other bond		
			Residues	Types	Bond length (Å)	Residue	Types	Bond length (Å)
Celocoxib	−7.7	8	GLY114	Conventional	2.18906	SER94, PHE219, PRO145, LEU216, PHE219, LEU95, PRO223	Halogen (Fluorine)	3.69134 5.10359 4.27206 4.7751 5.33003 5.37399 5.42432
							Pi-Pi T-shaped	
							Alkyl	
							Alkyl	
							Pi-Alkyl	
							Pi-Alkyl	
							Pi-Alkyl	
Ketoprofen	−7.5	3	TYR113	Conventional	2.11493	PHE219, PRO223	Pi-Pi T-shaped	4.78978 4.19071
							Pi-Alkyl	
D-pinitol	−5.0	3	ASP112, TYR113, GLY114	Conventional Conventional Conventional	2.68819 1.98643 1.92823	-	-	-

## 4 Conclusion

DPL exerted anti-inflammatory effect with drug-like properties. In our *in silico* study, DPL showed significant binding affinity with COX-2 with three conventional hydrogen bonds. We observed that the DPL binding residue and binding pocket of COX-2 are almost identical to the COX-2 inhibitor celecoxib binding residue and binding pocket of COX-2. We also found that the ketoprofen-COX-2 interaction binding pocket is similar to that. COX-2 pocket residues GLY114, TYR113, ASP112, THR11, TYR110, PHE219, LEU95, PRO223, and LEU116 are common in d-pinitol-COX-2 binding interactions and celecoxib-COX-2 binding interactions which provide that there is a possibility that DPL asserts its anti-inflammatory effect through inhibiting COX-2. Therefore, the ADME properties of DPL estimate good drug-like properties with highly water-soluble and fair bioavailability. We suppose DPL may act its anti-inflammatory effect through inhibiting COX-2 enzyme. Further studies are needed to claim its exact molecular interactions for its anti-inflammatory effect.

## Data Availability

The original contributions presented in the study are included in the article/Supplementary Material, further inquiries can be directed to the corresponding authors.
